# Plasma-Textured Teflon: Repulsion in Air of Water Droplets and Drag Reduction Underwater

**DOI:** 10.3390/biomimetics2010001

**Published:** 2017-01-23

**Authors:** Rosa Di Mundo, Francesco Bottiglione, Michele Notarnicola, Fabio Palumbo, Giuseppe Pascazio

**Affiliations:** 1 Department of Mechanics, Mathematics and Management, Politecnico di Bari, v.le Japigia 182, 70126 Bari, Italy; francesco.bottiglione@poliba.it (F.B.); giuseppe.pascazio@poliba.it (G.P.); 2 Department of Civil and Environmental Engineering and Chemistry, Politecnico di Bari, via Orabona 4, 70126 Bari, Italy; michele.notarnicola@poliba.it; 3 CNR-NANOTEC, via Orabona 4, 70126 Bari, Italy; fabio.palumbo@cnr.it

**Keywords:** Teflon, *Salvinia*, superhydrophobic, air-retaining, drop impact, air plastron, drag reduction

## Abstract

A superhydrophobic behavior can be obtained by properly modifying the surface topography of Teflon or other fluorinated polymers having an inherent hydrophobic character. According to this strategy, we have micro/nanotextured Teflon both as plane material (sheets) and as three-dimensional (3D) object (spheres) with a single step plasma process. The obtained textured Teflon samples were compared with those made of pristine Teflon in air, in terms of repulsion of impacting water droplets, and underwater, in terms of air layer behavior under static and dynamic conditions. The latter case was investigated by subjecting the spheres to a vertical fall in water. Modified surfaces present nanofilaments on the top of micrometric vertical structures, which can increase the air retaining capacity, resulting in a biomimicry effect due to a similarity with the *Salvinia molesta* leaf. On this surface, repulsion of impacting water droplets can be as fast as previously reached only on heated solids. Also, the air layer over the modified spheres underwater is shown to play a role in the observed reduction of hydrodynamic drag onto the moving object.

## 1. Introduction

Although known since the studies of Wenzel, and Cassie and Baxter [1,2], the attention towards the theme of superhydrophobicity was steeply renewed when the behavior of the lotus leaf was discovered and correlated with its multiscale structured hydrophobic (hence superhydrophobic) surface [3].

So far, this phenomenon has been investigated mainly for self-cleaning applications (textiles, windshields, etc.) [4] or microfluidics (lab on a chip) [5]. Self-cleaning applications require both the capability of favoring drop sliding (to carry out dirt particles) and that of repelling impacting water droplets (so the surface gets quickly rid of the liquid) [6].

A drop impacting on a superhydrophobic surface will spread out to a maximum diameter and then recoil to such an extent that it completely rebounds and leaves the solid material. In the past decade, it has been shown that the quasi-elastic bouncing of water drops is a typical characteristic of markedly superhydrohobic surfaces, and measurements of the time the drop remains in contact with the surface has been reported [7,8]. Since then, many works have been published on the ability of superhydrophobic surfaces of promoting bouncing of water droplets [9,10,11,12,13,14].

Only in the very last years, the skin friction drag reduction potentialities of superhydrophobic surfaces in macrofluidic applications have come under study. Experiments inspired by “water striders”—insects with superhydrophobic legs [15]—have been conducted with small superhydrophobic objects moving horizontally on water surface under laminar conditions. Important drag reduction (increased speed) results, though with very different extent, have been reported in this case (25%–70%) [16,17]. A reduction of drag has been observed also onto relatively larger ship-shaped objects moving on the water surface [18].

On the other hand, when the motion is tested underwater (vertical or horizontal direction) the results appear conflicting, since increased [16] and reduced drag [19,20,21] can be found in literature.

These clues indicate that the drag reducing performance underwater presents some critical aspects which deserve to be further investigated. 

Under this perspective, the inspiration of the *Salvinia molesta* leaf—aquatic plant capable of retaining air under water among the so called “eggbeater trichomes”—may represent an interesting strategy [21,22,23]. However, it is worth pointing out that such a bio-example works in nature strictly under static conditions and the way it can be improved and made suitable for dynamic conditions still represents an open challenge.

We have previously shown that the Cassie state, characterized by high static contact angle and low hysteresis under motion (i.e., low energy of adhesion between the solid and water) can be artificially obtained on several kinds of materials. In particular, several plastic materials have been turned to water repellent with a simple single step plasma etching process producing uniform expanses of nanometric and size-tunable ripples [24,25]. 

This process has also been shown to be effective in imparting another biomimetic functionality (i.e., the low reflectivity of visible and UV light, according to the moth-eye effect [26]).

Lately we have tested this process on Teflon (polytetrafluoroethylene, PTFE) in order to exploit the intrinsic hydrophobic properties of this polymer, hence utilizing simple ablation/roughening processes without the necessity of making coatings. This strategy has been also followed by other authors who have used a method of sandpaper sanding process, which is very cheap but less controllable [27].

We have shown that the repulsive properties against impacting water droplets (i.e., the critical pressure for water penetration) can be enhanced on the plasma modified material [28]. Also, the topographical modification has been shown to be tunable not only in terms of size but also in terms of shape, by ranging from spheres- to filaments-topped structures.

In this work, we move towards an in-water characterization of Teflon objects modified with such a plasma process. By making a comparative characterization between the untreated polymer and the best performing textured Teflon, we investigate the response in ambient air to impacting water droplets and, underwater, the appearance of trapped air over the surface under static and dynamic conditions. This way we aim at reaching a first understanding of the anti-drag potential of this superhydrophobic modification onto simply-shaped three-dimensional (3D) objects in water.

## 2. Materials and Methods

### 2.1. Teflon Surface Modification

Teflon 0.5 mm thick sheets, purchased from Goodfellow (Huntingdon, UK), were cut in 15 mm × 40 mm pieces. The Teflon spheres (solid spheres, 19 mm diameter, 2200 kg/m^3^ density) were purchased from New Ball SAS (Milan, Italy). Both samples types were sonicated in isopropyl alcohol and dried before processing. 

**Figure 1:**
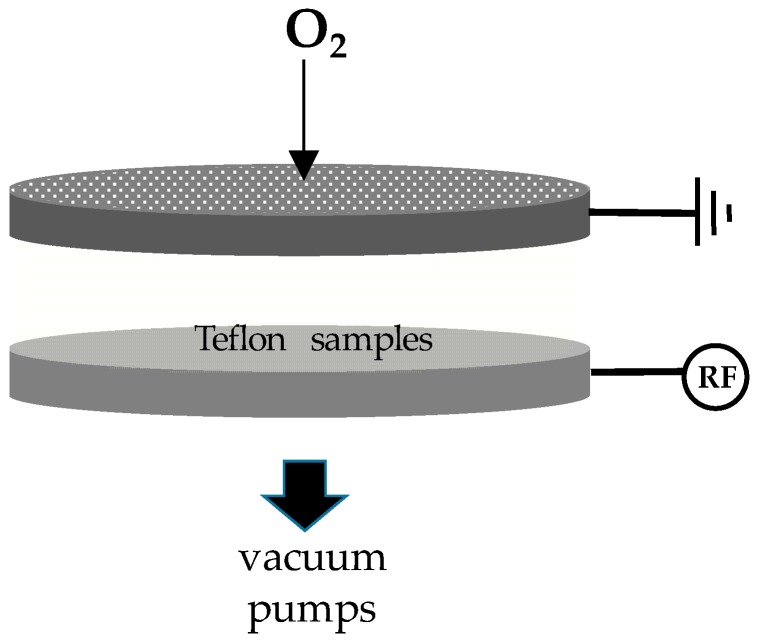
Scheme of the electrodic system of the plasma reactor set up for the plasma nano/microtexturing of the Teflon samples. The upper electrode is a shower-head type to allow homogeneous feed gas (O_2_) admission. Samples are placed on the bottom electrode which is radio-frequency (RF) powered.

The plasma micro/nanotexturing process was carried out in a home-made capacitively coupled reactor (Figure 1) with two parallel stainless steel electrodes having a gap of 4 cm, the upper one connected to the ground and the lower one connected to a radio-frequency (RF, 13.56 MHz; Caesar Dressler, Metzingen, Germany) power supply via a matching network. Both electrodes had a 314 cm^2^ surface area and were included in a stainless steel vacuum chamber evacuated with a rotative-turbomolecular pumping system (base pressure 10^−3^ Pa; Leybold GmbH, Cologne, Germany). Samples were located on the bottom electrode (the spheres were positioned within small steel rings and turned upside down at half-time process). Oxygen was used as feed gas. The gas flow rate was controlled by means of electronic gas flow meters, and vacuum measured with a Baratron gauge (MKS Instruments, Andover, MA, USA). Samples for this work were prepared feeding the chamber with 30 sccm of oxygen at 13 Pa, at a power of 200 W and a duration of 10 min.

### 2.2. Topographical and Chemical Characterization

The surface morphology of Teflon samples was investigated by means of field emission gun– scanning electron microscopy (FEG–SEM; Supra 40, Zeiss, Oberkochen, Germany) on specimens previously coated with 20 nm gold layer (Q150T sputter coater, Quorum Technology, Laughton, East Sussex, UK). Analysis were carried out at an extraction voltage of 3 KV, both at 0° and 45° tilting angle.

X-ray photoelectron spectroscopy (XPS) analyses were carried out by means of a PHI Versa Probe II Spectrometer (Physical Electronics, Chanhassen, MN, USA) with a monochromatic Al Kα X-ray source (1486.6 eV) at a spot size of 100 μm corresponding to a power of 100 W at a take-off angle of 45°. The C1s signal for C–C(H) bonds, with a binding energy of 284.7 eV, was used as an internal standard for the correction of the samples charging. Multipak software (Physical Electronics) was used to process acquired spectra.

### 2.3. Water Contact Angle and Drop Impact Measurement

Advancing and receding water contact angles (WCA) were measured by means of a home-made digital goniometer (PCO 1200h charge-coupled device (CCD) camera (PCO AG, Kelheim, Germany) combined with zoom optics and background lighting) on the plane materials. Advancing and receding angles were measured with the sessile drop technique by inducing (de-ionized water) drop volume variations from 1 to 4 μL at a speed of 0.5 μL/s in a microsyringe (Hamilton, 700 series with metallic needle of internal diameter of 0.127 mm; Postnova Analytics GmbH, Landsberg am Lech, Germany): the advancing angle is the maximum observed during the droplet growth; the receding one is measured just before the observation of the contact surface reduction during droplet volume decrease. 

Imaging of the impact was performed with the same system used for the WCA measurements. In this case the PCO 1200h camera was used in high-speed mode (1000 frames/s) and the de-ionized water drops (constant volume of 5 μL) were released from a microsyringe (Hamilton, 700 series with needle internal diameter of 0.127 mm; Postnova Analytics GmbH) held at a vertical distance (z) of 100 mm. Three repetitions were conducted per sample. The reported impact velocity was determined by measuring variation of drop position in the last five frames (5 ms) before the impact. The whole impact event was acquired, including drop release, impact with surface, bouncing, and (at least) the first landing on the surface. Image sequences were analyzed by ImageJ software [29] in order to evaluate contact/non-contact state of the drop (so to measure the contact and non-contact time) as well as to measure the time-variations of the contact length.

### 2.4. Optical Imaging of Air Layer on Submerged Surfaces

The digital portable microscope Dino Lite AM 4113T (AnMo Electronics Corporation, Taipei, Taiwan) was used to visualize the surface of the Teflon sheet samples when submerged by water. The microscope, fixed to a holder, was directed towards the sample attached to the ground of a transparent plastic box, at ca. 1 cm distance from the wall. Depth of water over the sample was 1 cm. Water was poured very slowly by increasing its level at a velocity of 1 cm/min. During acquisition, in order to maximize reflective effects due to air layer, the white small light-emitting diode (LED) lamps mounted on the head of the microscope were turned on, as well as a light diffuser placed at an angle of ca. 100° with the optical path. The acquisition was performed within one hour from the submersion. During this time no variation was observed on the submerged surfaces. 

### 2.5. Vertical Fall in Water Experiments

In order to evaluate the influence of the superhydrophobic treatment on hydrodynamic drag exerted during vertical fall in water, treated and untreated Teflon spheres (diameter 19 mm) were dropped into a tank 120 cm deep filled with tap water. Six spheres (three untreated and three treated) were released separately (zero initial velocity) in the tank from a distance of 55 mm from the water surface by using a mechanical release system with sliding wedges. The spheres were located over the aperture of the wedges where a groove had been made to ensure a still position before departure. The entire fall in the tank was recorded with a Canon E600D camera (Canon Italia S.p.A., Milan, Italy) at a rate of 50 frames/s. The acquired sequences were then analyzed with the open source software Tracker Video Analysis and Modelling Tool [30] in order to trace the sphere displacement vs time.

## 3. Results and Discussion

### 3.1. Effect of Plasma Modification: Chemical and Topographical Features 

**Figure 2:**
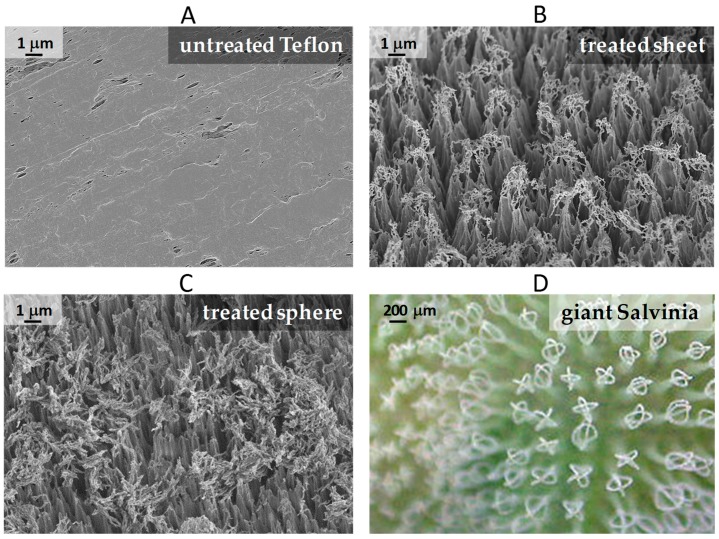
Scanning electron microscopy images of: (**A**) Untreated Teflon; (**B**) Surface-textured Teflon sheet at 45° tilt; (**C**) Surface-textured Teflon sphere at 45° tilt; (**D**) Micrograph of the giant salvinia (*Salvinia molesta*) surface [31].

In Figure 2, the SEM image of untreated Teflon is reported along with those of the plasma-modified Teflon sheet and sphere. Untreated Teflon (both as sheet and as sphere) show a surface relatively flat with sparse micrometric crevices.

The plasma-modified sheet surface appears very different: it presents a dense distribution of slender micrometric cusps on the apex bearing filaments with a width of few tens of a nanometer. The mean height and inter-distance of the vertical cusps are 5.5 ± 1.2 µm and 1.5 ± 0.5 µm, respectively. A pronounced irregularity is visible in size, position, shape of the structures, in agreement with the stochastic nature of the texturing process. In this process, indeed, a nanomasking is generated stochastically by the process itself on the substrate: during the discharge metal nanoparticles are sputtered from reactor electrodes and chamber walls and those deposited on the substrate act as etching masks since metals, contrary to the polymeric substrate, are not etched in oxygen plasmas. 

The texture on the sphere is quite similar. In particular, the vertical cusps present the same mean height and just a slightly lower interdistance; the filaments on the apex are very similar too, except for appearing more randomly distributed. A good similarity exists between the treated surfaces of these two substrates in spite of their different volume and height in the reactor (sphere diameter is 19 mm, sheet thickness is 1 mm).

**Figure 3:**
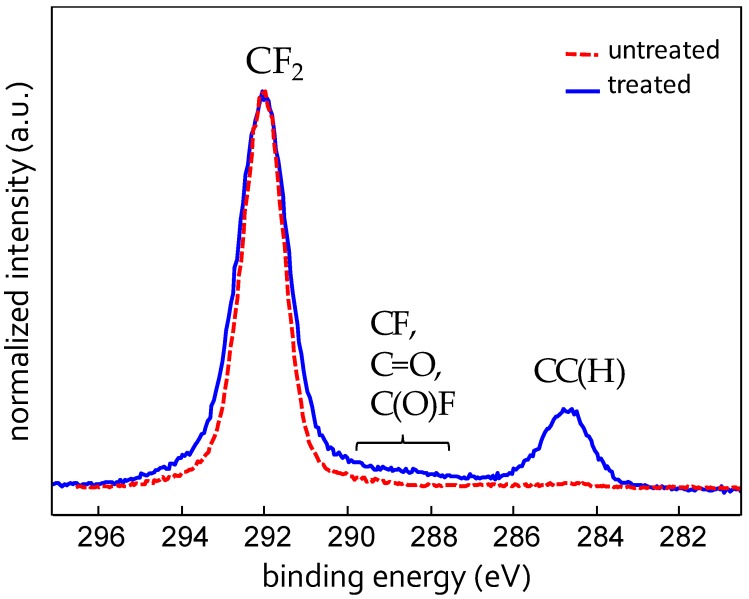
X-ray photoelectron spectroscopy (XPS) high resolution spectrum of the C1s signal for untreated- and plasma-treated Teflon sheet. a.u.: Arbitrary units.

X-ray photoelectron spectroscopy analysis has revealed that on the modified surface C, F, O, and Fe are present. The atomic percentages are reported in Table 1 along with those found on untreated Teflon, where, instead, only C, F, and O could be detected. The presence of iron on the modified surface is due to the above mentioned nanomasking mechanism. Figure 3 shows the C1s XPS signal of the compared samples. The C1s signal of the plasma modified surface presents a predominant contribution of the CF_2_ group (291.7 eV), a small contribution of the C–C(H) (284.7 eV) and very low components between 288 and 290 eV (not unambiguously ascribed to C–F, C=O, C(O)F and eventually other fluorine and/or oxygen containing groups). The C1s of untreated Teflon, instead, is made only of CF_2_ groups, as expected. The increase of the C–C(H) contribution in the modified sample indicates the cleavage of C–F bonds with subsequent increase of C–C units, also confirmed by the slightly reduced total percentage of F on this surface. These results indicate that under the investigated conditions the plasma process produces a slight chemical modification of the pristine Teflon, while, as seen above, the topographical modification is substantial. 

In Table 1, the advancing and receding WCA values are also reported: absolute values of the treated surface are steeply higher and show very low hysteresis. These values indicate a surface that is not extremely superhydrophobic, even when compared with surfaces obtained from processes similar to that here utilized, showing WCAs higher than 170° [25,28]. This is likely the consequence of a relatively high solid fraction [2] within the composite interface generated by the presence of these closely packed filaments.

### 3.2. Repulsive Behavior against Impacting Water Drops

**Figure 4:**
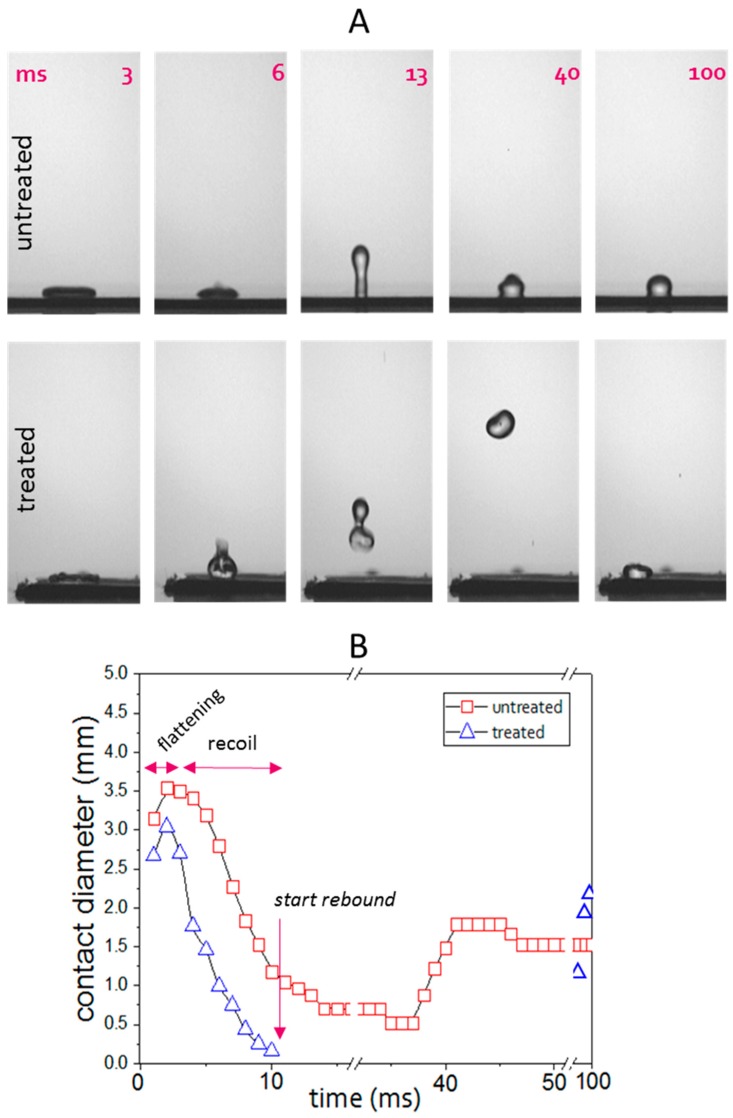
Drop impact on untreated and plasma treated plane Teflon: (**A**) Snapshots of a 5 µL water drop impacting onto the surfaces grabbed at the same times counted from the frame (0 ms) before the first contact; (**B**) Measured length of the surface-drop contact during the whole sequence.

The time lapse of a water drop released from a height of 100 mm and impacting at the speed of 1.43 m/s on the untreated Teflon and on the treated surface is reported in Figure 4A where the number on each frame indicates the elapsed time in ms from the frame before the first contact. In Figure 4B, we report a diagram with the length of the drop-surface contact (contact diameter) as a function of time. 

The impact regime of a liquid drop hitting a solid surface can be classified according to the non-dimensional $W_{e}$ number, which is uniquely a function of the drop features ($W_{e}=\rho v_{imp}{}^{2}r/\gamma$, with ρ, γ, $v_{imp}$, r, being respectively the water density and surface tension, impact velocity, spherical drop radius). The impact speed of 1.43 m/s, given the drop volume of 5 μL, gives $W_{e}$, which indicates an impact regime of medium/high deformation [7,8,9,10,11,12,13,14,15,16,17,18,19,20,21,22,23,24,25,26,27,28,29,31,32]. It should be noted that this value of $W_{e}$ is considered consistent with that of light rain [33]. 

On both surfaces a first stage of flattening and a successive stage of fast recoil are observed. On the untreated one the recoil is slightly slower and stops at a non-zero contact length. This leads to the separation of a bigger fragment from a smaller one, which remains always attached to the surface. Thus, a full rebound is not observed on this surface. On the other hand, the plasma modified surface gives rise to a full steep rebound which starts just after 9.7 ± 0.4 ms of contact. 

We note that by dividing this time value by the inertial-capillary timescale $\tau =\sqrt{\rho r^{3}/\gamma }$ [34] which in our conditions equals 4.04 ms, a non-dimensional t* = 2.4 is obtained. The dimensionless time t* allows to compare our data with those presented in literature for different drop sizes both in the case of low and large deformation regimes ($W_{e}$ < 1 and $W_{e}$ > 1). These literature examples have been recently reviewed in [34] where it can be easily seen that t* = 2.4 is reported only for water drops hitting a hot solid. In the case of room temperature surfaces, only the addition of macrostructures/ridges can further abate the contact time on a superhydrophobic surface [34,35]. Longer contact times have been also reported in our previous work on sphere-on-cone type textured Teflon surfaces [28].

In Figure 4 we report all the sequence for including the whole rebound stage until the second landing of the drop (98 ms). This highlights the ratio between the time the droplet spends in contact with the surface and the time of flight after the rebound. The ratio between these time amounts is inversely correlated to the probability that wetting, sticking, icing, dirtying phenomena take place and provide a fast idea of the surface ability to keep itself rid of the liquid. 

We also notice that a big difference exists between the compared surfaces even in the flattening and the recoil stages: by observing the frames at 3 ms (flattening) and 6 ms (recoil) within the liquid in contact with the treated surface, a certain perturbation can be appreciated. This effect of perturbation without fragmentation has been already observed onto other nanostructured superhydrophobic materials under similar impact conditions [34].

The perturbation of the liquid in contact with the surface as well as the very short duration of the contact as discussed above, can be ascribed to the strong attitude of the treated surface to repel incoming high velocity water. 

### 3.3. Appearance of Still Surfaces Underwater

**Figure 5:**
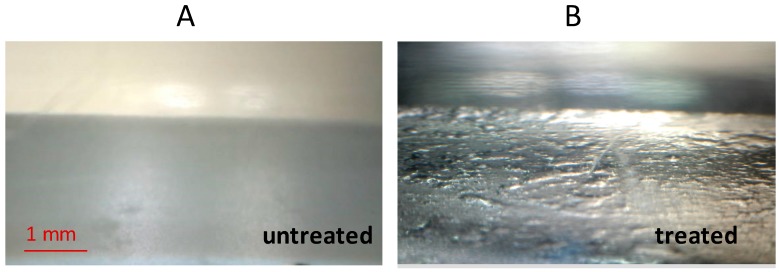
Underwater optical microscopy images of still (**A**) untreated and (**B**) treated Teflon. Samples are fixed to the ground of a transparent box filled with 1 cm deep water.

The appearance of these surfaces when submerged by water is shown in the optical photograph of Figure 5. When air is present at the Teflon–water interface, the transition of incident visible light through two means with different refractive index, water and air, gives rise to a specular reflectance, making metallic the appearance of the surface. This is often called “silvery sheen” and some examples are reported in literature [16,22,36,37]. While on the untreated Teflon surface, no shine is observed and the sample appears just like when placed in air, in the case of the treated surface a markedly shiny layer is present. The profile of the air layer seems to form sub-millimetric areas with a deeper thickness, like larger bubbles over an extended bed of air. The shiny layer on this surface is more pronounced than that observed onto other Teflon-structured surfaces that are even more superhydrophobic (higher WCA) than this one [38].

This means that this surface has a stronger ability to favor retention of air against wetting events. Since not extremely superhydophobic, it is likely that this ability arises from a certain capacity to trap amounts of air beneath its structures.

This function is probably exerted by the covered voids, which are formed by the bending of the filamentary heads. This concept resembles what is generally ascribed to the surface of the water fern giant salvinia *Salvinia molesta* (refer to Figure 2 for comparison), where eggbeaters-like structures act as reservoir of air underwater, though the shape and the size scale is much different (bigger in the case of *Salvinia*).

It has been observed that *Salvinia* can stay dry for several days when placed underwater, though it is not particularly superhydrophobic [23]. From the microscopy images in Figure 2 it can be appreciated that for plasma-treated samples the distance between the vertical cusps (pillars) l is of the order of micron units (1.5 µm is the measured mean), the distance between filaments, instead, is of the order of tens nanometers (l≈0.02 µm). Overpressure in the liquid due to several causes (capillary pressure, impact dynamics, hydrostatic pressure) may lead the water to fill the inter-pillar spaces, but a much higher pressure would be necessary to also fill the cavities beneath the filaments. Indeed, given that the pressure P to fill a cavity scales like P~1/l [28] it can be calculated that the pressure to penetrate beneath the filaments structure is 100 times that necessary to invade the inter-pillar spaces. 

### 3.4. Drag Variation on Teflon Sphere Falling in Water

**Figure 6:**
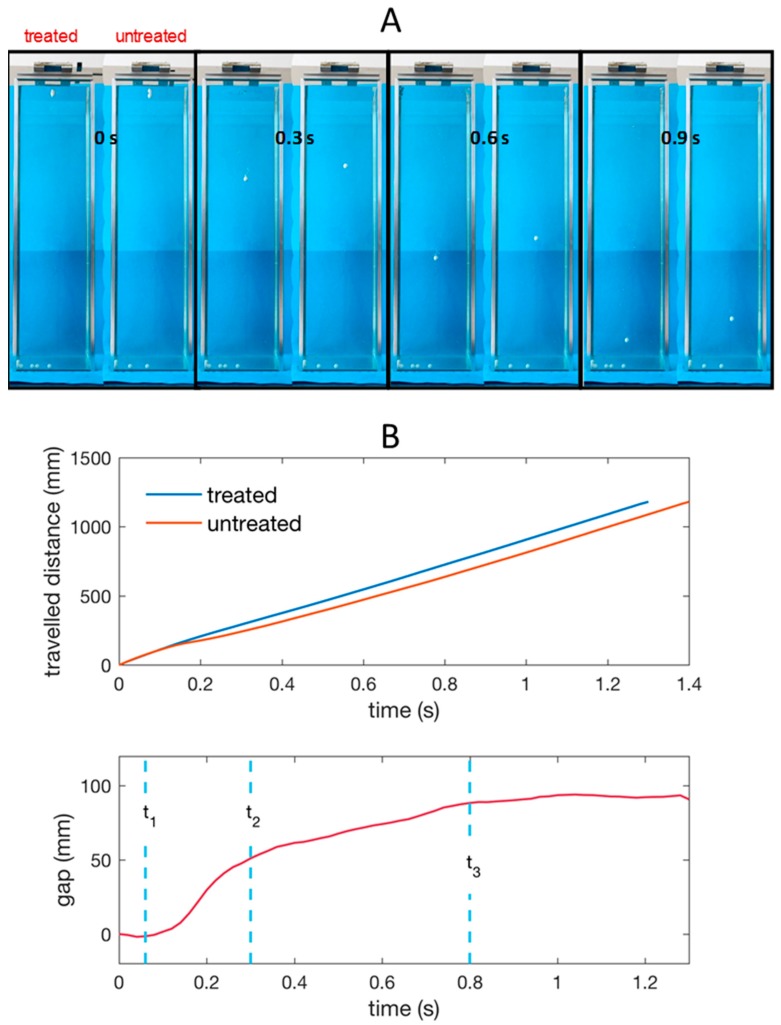
(**A**) Snapshots of the untreated and treated Teflon spheres falling into the water tank recorded at different instants after the start (water entry); (**B**) Vertical coordinate *z* (depth travelled under the water free surface) of the untreated and treated sphere and corresponding gap vs time. The times t_1_, t_2_, and t_3_ are those corresponding to the images in Figure 7.

In Figure 6 we show some frames of the spheres’ fall by reporting the treated and the untreated one grabbed at different instants after the start (water entry). The spheres were released from a distance of 55 mm from the water surface, so the velocity of the sphere entry (impact) in water is about 1 m/s. 

It can be noted that the superhydrophobic sphere gets ahead with respect to the untreated one since the first trait and holds the vantage until the end of the fall.

The time interval between the water entry and the spheres impact with the bottom of the tank is Δt=1.38±0.02 s for the untreated sphere and Δt=1.32±0.02 s for the treated sphere, leading to an average velocity calculated as v=Δz/Δt of 0.86 m/s for the untreated and 0.89 m/s for the treated sphere (Δz=1.18 m). 

A rough comparison of the performance of the treated and untreated spheres is achieved considering the ratio of the average velocities squared, that, in the Reynolds regime of the experiment (up to $Re\approx 1.6-1.7\times 10^{4}$) equals the inverse ratio of the average drag coefficients ${\bar{C}}_{D}{}^{tr}/{\bar{C}}_{D}{}^{un}={\left({\bar{v}}^{un}/{\bar{v}}^{tr}\right)}^{2}\approx 0.93$. 

Other authors [21], testing glass spheres coated with metal-oxide particles in a polymer matrix, or investigating acrylic spheres coated with Granger’s Gore-Tex [19], reported similar results.

**Figure 7:**
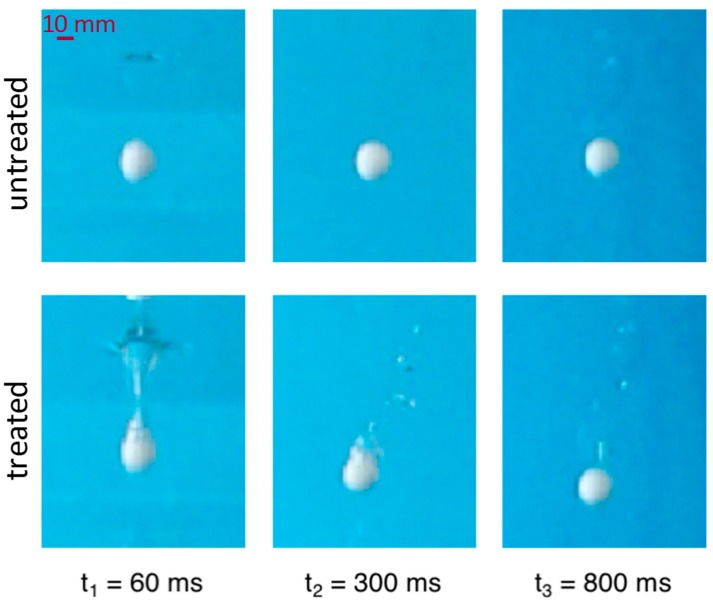
High magnification images of the spheres falling in the tanks at some representative times (t_1_, t_2_, and t_3_ shown in Figure 6 diagrams).

However, we note that the mean velocity related to the whole liquid depth gives only partial information since a non-uniform variation of the relative position of the spheres is observed along the path. Indeed, Figure 6B shows the difference (gap) between the vertical coordinates *z* (depth under water free surface) of the two spheres vs time. Very small deviations from this curve have been observed (in the first trait curves fully overlap and in the end deviate at most of four percent) on the three repetitions conducted. After a short time $t_{1}=60\;\mathrm{ms}$, the depth is equal for the two spheres. In Figure 7 it is shown that at the end of this first time interval the treated sphere has generated an air cavity, which is completely absent on the untreated sphere. At $t_{1}=60\;\mathrm{ms}$ the air cavity is sealing on the top and separating from liquid-free surface. 

Such behavior of bodies with superhydrophobic coatings is reported in works focused on dynamics of their impact with the liquid [39,40]. 

The air volume adheres to the treated sphere, which, in the subsequent phase from $t_{1}=60\;\mathrm{ms}$ to $t_{2}=300\;\mathrm{ms}$, falls enclosed in a sort of spindle-shaped “air capsule”. This phenomenon is not observed on the untreated sphere, which is unable to retain air in any form. In this second time interval, from $t_{1}$ to $t_{2}$, (Figure 6) one can observe that the treated sphere overcomes the untreated sphere very quickly, meaning that the overall drag force on the treated sphere is much less then on the untreated one in this phase. 

Indeed, if one considers that in the treated sphere the upward force due to buoyancy is increased because of the volume of the retained air, the increased falling down velocity reveals that the drag has been reduced, well beyond compensation of the buoyancy effect. At time $t_{2}=300\;\mathrm{ms}$ the treated sphere starts losing the air spindle (Figure 7); air bubbles detach and move upwards and the volume of the air capsule gradually decreases. No change is visible on the surface of the untreated sphere. Figure 6 (bottom) shows that in this second phase the difference of depth between the two spheres changes less steeply, meaning that the relative velocity is decreasing. At time $t_{3}=800\;\mathrm{ms}$ the treated sphere seems to lose the air capsule, or at least the macroscopic portion (Figure 7). Afterwards, the gap does not change anymore, indicating that the velocity of the two spheres is the same. We have also calculated that the velocity in this phase is almost constant for both the spheres (terminal velocity $v_{T}$) and equals 0.91 m/s. The velocity resulting from balancing gravity, buoyancy and drag in steady-state conditions was calculated by solving the falling sphere dynamics equation, considering a Reynolds dependent drag coefficient as previously reported [41]. We obtain $v_{T}\approx 0.83\;m/s$ with $C_{D}\approx 0.43$.

Thus, this superhydrophobic treatment has substantially reduced the drag in that phase of the sphere fall when the spindle-shaped air capsule is well attached to the surface. Indeed, the ratio ${\bar{C}}_{D}{}^{tr}/{\bar{C}}_{D}{}^{un}={\left({\bar{v}}^{un}/{\bar{v}}^{tr}\right)}^{2}$ restricted to the period 0–800 ms equals 0.77, much lower than that extended to the whole path above reported.

In agreement with previous predictions [42], in the Reynolds regime of our experiments, a sufficiently thick air layer can change the separation points and the wake cross sectional area (spindle shape), thus modifying the overall drag substantially. 

## 4. Conclusions

The surface of Teflon can be modified with a single step plasma etching process generating micrometric cusps with nanosized filaments structures having a chemical composition similar to pristine Teflon. This modification results in considerably different wetting effects compared to untreated material both in air and underwater.

In summary, we have shown that:

(i) Modified plane Teflon in ambient air repels impacting droplets at high velocity by promoting a full detachment of the liquid in a time as low as previously observed only on heated solids;
(ii) When placed underwater, a shiny film is observed on the treated surface, indicating the formation of a continuous air layer between the solid and the liquid;
(iii) The above-mentioned performances are more pronounced on this surface than on other previously shown textured surfaces with higher water contact angle, indicating a relatively higher retaining capacity though a relatively lower superhydrophobicity. These results recall a wetting effect which is similar to that of the giant salvinia leaf, which has a high air retention ability though not being particularly superhydrophobic. This may be correlated to the morphology generated by the plasma etching process, similar to the eggbeaters structures on that leaf, both characterized by pillars bearing a curved feature on the top, hiding, this way, the bottom part of the surface cavities.
(iv) Teflon spheres modified under the same conditions present a similar texture; when falling into water (laminar conditions) these samples show a higher mean velocity than the pristine ones. This effect slightly decreases in time (i.e., along the fall), with a rate which appears to be correlated with depletion of an “air spindle” connected with the superhydrophobic sphere.

Future experiments will be devoted to a more powerful imaging of the air plastron in order to yield a detailed quantitative analysis of the correlation between the sphere motion (drag resistance) and air plastron status. Furthermore, spheres made of different density and different diameter are being planned to be tested in order to investigate different flow regime conditions.

## Figures and Tables

**Table 1:** X-ray photoelectron spectroscopy (XPS) atomic percentage and dynamic water contact angle (WCA) values of the untreated and the plasma-modified Teflon samples.

Teflon Surface	C (%)	F (%)	O (%)	Fe (%)	Adv. WCA (°)	Rec. WCA (°)
Untreated	33	66	1	-	125	94
Treated	33	58	4	4	162	160

Adv.: Advancing; Rec.: Receding.
